# The chimeric Japanese encephalitis/Dengue 2 virus protects mice from challenge by both dengue virus and JEV virulent virus

**DOI:** 10.1007/s13238-016-0363-5

**Published:** 2017-01-25

**Authors:** Jian Yang, Huiqiang Yang, Zhushi Li, Hua Lin, Yu Zhao, Wei Wang, Shuai Tan, Xianwu Zeng, Yuhua Li

**Affiliations:** 1 Department of Viral Vaccine, Chengdu Institute of Biological Products Co., Ltd., Chengdu, 610023 China; 2 Department of Microbiology and Immunology, North Sichuan Medical College, Nanchong, 637007 China; 3 Department of Arboviruses Vaccine, National Institute for Food and Drug Control, Beijing, 100050 China; 4 State Key Laboratory of Biotherapy and Cancer Center, West China Hospital, Sichuan University and Collaborative Innovation Center for Biotherapy, Chengdu, 610000 China

**Dear Editor**

Dengue fever (DF) is the most significant emerging arbovirus disease, which is caused by four distinct serotypes of Dengue viruses (DENV-1 to DENV-4). Serotype-specific immunity does not confer cross-protection against secondary infections from heterologous serotypes, which leads to dengue haemorrhagic fever (DHF) and dengue shock syndrome (DSS) as a result of antibody-dependent enhancement (ADE) of infection that is mediated by non-neutralizing, cross-reactive antibodies (Kliks et al., 1989). The intense efforts to develop a safe and efficient DENV vaccine have involved using a variety of traditional biological technologies in the past 50 years (Durbin et al., 2005; McArthur et al., 2008), yet no commercial DENV vaccine has been generated until now.

Developing DENV vaccine has been a high priority of the World Health Organization (WHO) (Chambers et al., 1997). Both Dengue virus and Japanese encephalitis virus (JEV) belong to the Flaviviridae. The JE attenuated live vaccine (SA14-14-2) was licensed in 1989 in China. Its safety and effectiveness have been demonstrated in large scale clinical surveillance and practical application of 600 million doses for more than 20 years in China and in Southeast Asian countries including Korea, Thailand and India (Yu, 2010). No vaccine associated encephalitis cases were reported for this JE vaccine, compared with 21 encephalitis cases associated with vaccination with the yellow fever (YF) vaccine 17D which is regarded as a safety vaccine (Monath et al., 1999). In particular, its unique profile of attenuated neurovirulence stability ensures the safety of the JE live vaccine and enables the utility of this live vaccine strain as a vector to develop vaccines against other Flaviviruses. This strategy has become feasible with the advent of reverse genetic method that has been used to construct chimeric flaviviruses (Pletnev et al., 2002; Mathenge et al., 2004). This approach has been used to prepare chimeric viruses YF/DENV (1–4) and such viruses exhibit high immunogenicity and low virulence (Chambers et al., 2003; Guirakhoo et al., 2004).

In this study, the prM/E genes of JEV SA14-14-2 in the plasmid pACNR-JEV were replaced with the prM/E gene from the DENV-2 PUO-218 strain to generate the plasmid pACNR-JE/DENV-2 with the method reported by Li and Yang (Li et al., 2014; Yang et al., 2016). Results show that the restriction enzyme digestion of pACNR-JE/DENV-2 yielded the DNA fragments that were resolved by electrophoresis as expected. The chimeric virus JE/DENV-2 was produced by transfecting viral RNA into the BHK21 cells. The titre of the chimeric virus was approximately 3.3 log_10_PFU/mL. The size of the plaques ranged from 1 to 2 mm in diameter, which was smaller than that of JEV SA14-14-2 (2 to 3 mm in diameter) but larger than that of the DENV-2 (0.5–1 mm in diameter) (Fig. 1A). The results indicate that the chimeric virus is more like dengue virus rather than JEV and it is possible that the prM/E determines the size and formation of the plaque (Li et al., 2013).

**Figure 1:**
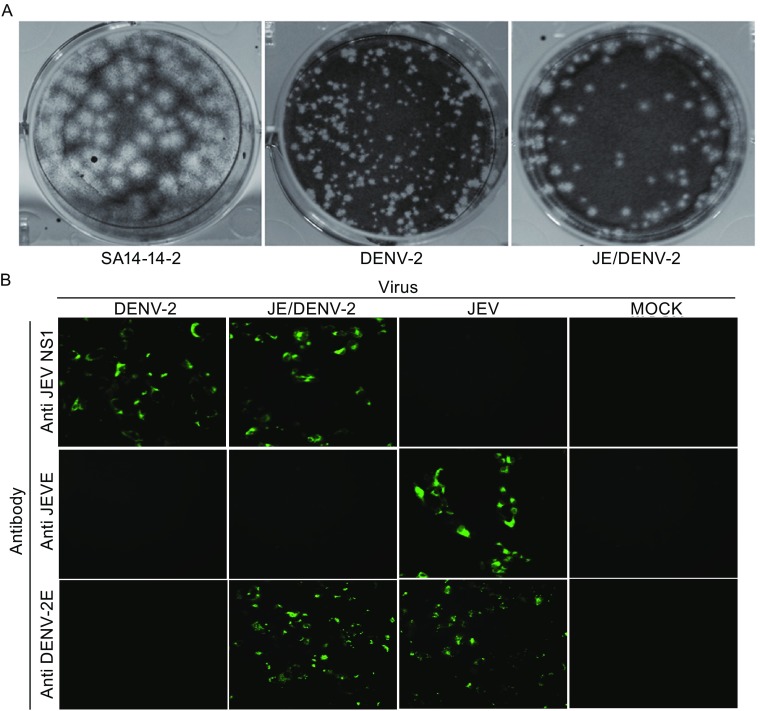
**The diameter of plaque and viral proteins expression of JEV, JE/DENV-2 and DENV-2 in BHK21 cells**. (A) The formation and diameter of plaques in BHK21 cells. (B) BHK21 cells were infected with each of these three viruses. Immunofluorescence staining was performed using antibodies recognizing DENV-2 E protein (1:10 dilution, top panel), JEV E protein (1:10 dilution, mid panel), or JEV NS1 protein (1:10 dilution, bottom panel). Mock represents no virus infection. DENV, dengue virus; JEV, Japanese encephalitis virus; JE, Japanese encephalitis; E, envelope protein; NS: non-structural protein

As a vaccine candidate, it must be stable in passaging process. Sequence of the produced chimeric viruses from passage 1th to passage 14th was determined, which all contained the 10,959 nucleotides in the complete genome of the engineered JE/DENV-2 virus. No mutation was detected prior to passage 10th. 4 mutations were detected from passage 11th to 14th. They are at nucleotide position 39 (A to T) in the 5′ UTR, position 1,531 (C to T) in the structural protein E region resulting in a serine to phenylalanine mutation, position 3,511 (A to T) in the non-structural protein region resulting in a histidine to leucine mutation, and position 3,468 (A to C) in the non-structural protein region resulting in a methionine to leucine mutation.

Indirect immunofluorescence staining was adopted to detect the expression of viral proteins from chimeric virus JE/DENV-2 and the parental viruses JEV SA14-14-2 and DENV-2. The results showed that monoclonal antibody against the DENV-2 E protein detected the expression of the DENV-2 E protein in BHK21 cells that were infected with the DENV-2 virus and chimeric virus JE/DENV-2, but not with JEV (Fig. 1B top panel). Monoclonal antibody against the JEV E protein detected the expression of JEV E protein only in BHK21 cells infected by the JEV SA14-14-2 virus (Fig. 1B mid panel). Monoclonal antibody against the JEV NS1 protein detected the expression of JEV NS1 protein in cells infected by JEV SA14-14-2 or JE/DENV-2, but not by DENV-2 virus (Fig. 1B bottom panel). These results confirm the successful expression of DENV-2 E protein from the chimeric virus JE/DENV-2 and indicate that the SA14-14-2 is a good vector to express exogenous antigen (Li et al., 2013; Li et al., 2014; Yang et al., 2016).

The growth kinetics of the JE/DENV-2 virus were determined by infecting PHK, Vero or C6/36 cells and the results were compared to the replication data of the DENV-2 virus NGC strain and the JEV SA14-14-2 virus. In PHK cells, the JE/DENV-2 propagated to lower titres at all time points compared to JEV which reached the peak titre of 7.3 log_10_PFU/mL at 48 h post-infection. Instead, JE/DENV-2 exhibited a replication profile that was similar to that DENV-2. The peak titres of JE/DENV-2 and DENV-2 were 4.5 log_10_PFU/mL and 5.4 log_10_PFU/mL at 36 h, respectively (Fig. 2A). In Vero cells, JE/DENV-2 grew more rapidly than DENV-2, yet the titres of JE/DENV-2 were generally low with a peak value of 3.7 log_10_PFU/mL in 120 h despite of the different conditions that we tested. In contrast, JEV SA14-14-2 reached a peak titre of 6.6 log_10_PFU/mL at 96 h post-infection. DENV-2 replicated very slowly at the early time points compared to JEV, but was able to reach a peak titre of approximately 6.2 log_10_PFU/mL at 120 h (Fig. 2B). In C6/36 cells, JE/DENV-2 propagated less efficiently than JEV and DENV-2, and reached its peak titre of approximately 4.8 at 48 h post-infection compared to the peak titre of 7.2 log_10_PFU/mL for JEV SA14-14-2 at 72 h and the peak titre of 6.0 log_10_PFU/mL for DENV-2 at 72 h (Fig. 2C). We also investigated the growth kinetics of JE/DENV-2 with different M.O.I. in PHK cells. The virus reached its peak titre of 4.9 log_10_PFU/mL at 24 h post-inoculation with the 0.025 M.O.I. and the peak titre of 5.5 log_10_PFU/mL at 48 h post-inoculation with the 0.005 M.O.I. and the 0.001 M.O.I. (Fig. 2D). In conclusion, the chimeric virus JE/DENV-2 propagates less efficiently compared to its parental virus JEV and exhibits a stronger or similar replication capacity compared to DENV-2.

**Figure 2:**
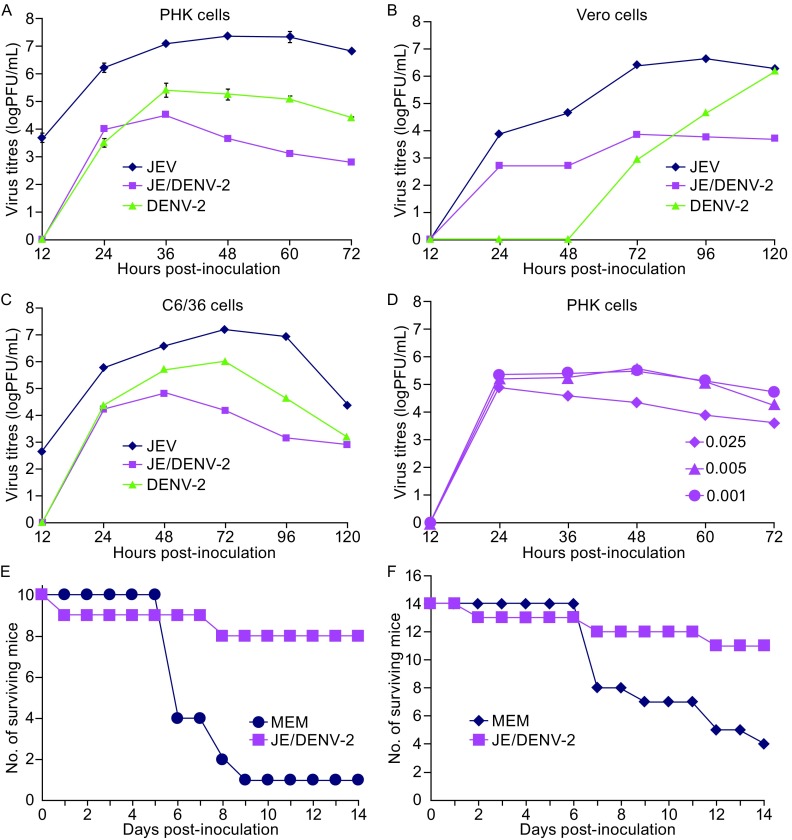
**The growth curve of the chimeric virus in PHK, Vero and C6/36 cells and the chimeric virus JE/DENV-2 confers protection against neuroadapted DENV-2 virus (New Guinea strain, NGC) and JE virulent virus JEV/SA14**. The growth curve of DENV-2, JE/DENV-2 and JEV in PHK cells (A), Vero cells (B), C6/36 cells (C) and PHK cells (D) (different M.O.I.). Mice were immunized with JE/DENV-2 via the i.p. route, or mock immunized (MEM). One month after immunization, mice were challenged via the i.c. route with neuroadapted DENV-2 NGC strain virus (shown in E) or via i.p. inoculation with JEV/SA14 virus (shown in F). The number of survived mice at each time point was recorded and plotted. M.O.I., multiplicity of infection; MEM, minimum essential medium; PHK, primary hamster kidney

Neurovirulence of the JE/DENV-2 virus was evaluated via the intra-cerebral (i.c.) route inoculation of 4-week old Kun-Min mice. The neurovirulence result showed that LD_50_ (log_10_PFU) of JE/DENV-2 was 2.7. The JE live vaccine strain SA14-14-2 was also tested as the control, which did not show any neurovirulence effect in mice. This result comes as a surprise, because both the parental viruses are avirulent to mice. No mouse died in the neuroinvasiveness test performed with JE/DENV-2 or SA14-14-2.

All mice used for immunogenicity study did not exhibit any sign of illness or mortality over the period of observation. The sera were treated for PRNT as described in other research. All mice immunized with the JE/DENV-2 chimeric virus were seroconverted to produce anti-DENV-2 antibody, based on the antibody titre value of 1:10. Antibody titre from each group was pooled to calculate geometric mean titres (GMTs). From week 1, 3, 5, 7, 9 and week 11 post boosting, the GMT reached 80, 92, 92, 80, 106 and 184, respectively. Differences of GMTs over this interval were not statistically significant.

We then investigated whether the JE/DENV-2 chimeric virus is able to induce protection against neuroadapted DENV-2 virus (NGC strain). For this purpose, mice were immunized with the JE/DENV-2 virus of 4.5 log_10_PFU via intra-peritoneal route (i.p.), followed by challenging via the i.c. route with the DENV-2 NGC strain of 4.5 log_10_PFU one month after immunization. The mice were observed for 2 additional weeks. None of these mice manifested any signs of illness for the one-month period before virus challenge. Results show that eight of nine immunized mice resisted the challenge of neuroadapted DENV-2 NGC strain, however, nine of ten mock-immunized mice succumbed to the challenge (Fig. 2E).

Because the chimeric JE/DENV-2 virus was constructed using JE SA14-14-2 as the vector, we suspected that mice immunized with JE/DENV-2 might also be protected from the challenge with JE virulent virus JEV/SA14, in which the prM/E gene of JEV SA14-14-2 was replaced with that of JEV wild type SA14. Some studies reported that antibody to JEV NS1 protein can provide immune protection against wild type JEV (Li et al., 2012). To test this possibility, mice were immunized with JE/DENV-2 and then challenged via the i.p. route with JEV/SA14 of 6.6 log_10_PFU one month after immunization. The mortality rate was 15% in the immunized group compared to 71% in mock-immunize group (Fig. 2F). The results are according with Li’s results (Li et al., 2013). Together, these data demonstrate that immunization of mice with the chimeric virus JE/DENV-2 elicits strong protection against infection by either neuroadapted DENV-2 or virulent JEV/SA14.

In summary, results of this study demonstrate that the JE live vaccine virus serves as a promising vector for engineering flavivirus vaccines. The chimeric virus causes neurovirulence to mice via i.c. inoculation although the parent virus is avirulent and the reason need to be elucidated in the following work, meanwhile the neurovirulence in primate of chimeric virus need to be studied. Most importantly, we found that JE/DENV-2 may induce immunity protection against not only DENV-2 but also JEV. This will provide new sight to design vaccine to against Flavivirus.

## **FOOTNOTES**

LYH and YHQ conceived and designed the studies. YJ, LZS, LH and ZY performed the experiments. ZXW contributed reagents, materials and analysis tools. YJ and LYH analyzed the data. YJ prepared the manuscript. We would like to thank Professor Yu Yong-xin for providing DENV-2 virus (NGC strain). This work was supported by the National High Technology Research and Development Program (863 Program) (No. 2012AA02A401). The funder had no role in study design, data collection and analysis, decision to publish, or preparation of the manuscript.

Jian Yang, Huiqiang Yang, Zhushi Li, Hua Lin, Yu Zhao, Wei Wang, Shuai Tan, Xianwu Zeng, and Yuhua Li declare that they have no conflict of interest.

The experimental protocols involving mice were approved by the Animal Experimental Committee of Chengdu Institute of Biological Products Co. Ltd. All institutional and national guidelines for the care and use of laboratory animals were followed.

## Electronic supplementary material

Below is the link to the electronic supplementary material.

Supplementary material 1 (PDF 127 kb)
