# Supplementary material for: The chimeric Japanese encephalitis/Dengue 2 virus protects mice from challenge by both dengue virus and JEV virulent virus
Source: Protein Cell. 2017 Jan 25;8(3):225–9. doi: 10.1007/s13238-016-0363-5 (PMC5326627; doi:10.1007/s13238-016-0363-5)

The chimeric Japanese encephalitis/Dengue 2 virus protects mice from challenge by both dengue virus and JEV virulent virus

Jian Yang, Huiqiang Yang, Zhushi Li, Hua Lin, Yu Zhao,

Wei Wang, Shuai Tan, Xianwu Zeng, Yuhua Li

Materials and Methods

Cells, viruses and animals

BHK-21 cells, Vero cells and C6/36 cells were purchased from ATCC. Primary hamster kidney (PHK) cells and JE live attenuated vaccine SA14-14-2 were prepared in Chengdu Institute of Biological Products Co., Ltd. DENV-2 virus New Guinea C (NGC) strain was obtained from National Institute for Food and Drug Control, P.R.C and passaged twice in suckling brain，then propagated once in C6/36 cells at 28℃ to prepare the working virus stocks. The virulent JEV/SA14 was constructed by replacing the prM/E gene of JEV SA14-14-2 with that of JEV wild type SA14. Kun Min mice were maintained in the animal center in Chengdu Institute of Biological Products Co., Ltd. All animal welfare, care, surgical, and research procedures are consistent with the Guide for the Care and Use of Laboratory Animals and were approved by the Institutional Animal Care and Use Committee of National Institutes for Food and Drug Control.

Construction of pACNR-JEV and pACNR-JEV/DENV-2

In order to generate the recombinant plasmid pACNR-JEV/DENV-2, we first prepared the pACNR-JEV plasmid that contains the full length clone of JEV SA14-14-2. Briefly, RNA of the JEV SA14-14-2 virus was extracted and reverse

transcribed into viral cDNA. Seven fragments were amplified to cover the whole genome of JEV SA14-14-2. Each fragment contains unique restriction sites for subsequent cloning. To facilitate plasmid construction, a silent mutation at nucleotide (nt) position 473 (A to C) within JEV protein C gene was introduced to create a KasI restriction site. These fragments were cloned into the pACNR vector to create the plasmid pACNRA-JEV. A SP6 promoter was added to the 5’ terminus of JEV full length DNA for in vitro transcription.

The pACNR-JEV/DENV-2 recombinant plasmid was constructed in two steps (Fig. S1): the 5' end plasmid and then the full-length plasmid. First, the TOPO-DENV-2 prM/E plasmid was engineered by inserting into the TOPO TA vector (Invitrogen) a synthesized fragment that contained the prM and E genes from DENV-2 PUO-218 strain (GenBank: D00345.1). This fragment had a KasI restriction site at 5' end and was followed by part of 5' terminal of NS1 genes of JEV vaccine strain SA14-14-2, but the last nine nucleotides at 3' terminus of JEV E gene were retained. The TOPO-DENV-2 prM/E plasmid was digested with KasI/BglII restriction enzymes to release the prM/E fragment of DENV-2, which was then subcloned into the pACNR-JEV 5' end plasmid to replace the counterpart of JEV. The resulting plasmid is named pACNR-JEV/DENV-2 5'. The 5' portion of chimeric virus JEV/DENV-2 cDNA (1-3428 nt) was released from the pACNR-JEV/DENV-2 5' end plasmid by digestion with AscI/BspEI, and subcloned into the pACNR-JEV full length plasmid to replace the counterparts of JEV, to create the pACNR-JEV/DENV-2 full length plasmid. Digestions with different restriction enzymes were performed to verify the

recombinant plasmid (Fig. S2). The JEV/DENV-2 full-length cDNA in the plasmid was sequenced to confirm the cloned viral sequences.

RNA transcription and production of chimeric virus

Plasmid pACNR-JEV/DENV-2 was linearized with XhoI digestion. The cohesive ends were removed using mung bean nuclease at 30℃ for 30 min. The plasmid DNA was then purified with PCR purification kit (Invitrogen). RNA was transcribed from the linearized plasmid DNA using a RiboMAX™ Large Scale RNA Production Systems SP6 kit (Promega) in the presence of Ribo m7G Cap Analog (Promega). The reactions were treated with DNase I (RQ1 RNase-free DNase, Promega) to remove plasmid DNA, followed by purification with Viral RNA Mini kit (Qiagen). 4μg RNA was transfected into 5×10^6 BHK21 cells by electroporation at 140 V and 25 ms using a Gene Pulser II apparatus (Bio-Rad). The transfected cells were cultured in Eagle's MEM media supplemented with 10% FBS. The cell culture supernatants were harvested 5 days after transfection when the cytopathic effects were observed. The culture supernatants were designated as the first passage of the chimeric viruses.

Viral plaque assay

Plaque assay for JE/DENV-2, JEV SA14-14-2 and DENV-2 viruses were carried out by infecting confluent monolayer of BHK21 cells. The confluent BHK21 cell monolayer was prepared with eagles' MEM plus 10% serum and incubated with viruses at 37℃ for 1 hr. After removing the inocula, the agarose (Takara Inc.) overlay in Eagle's MEM plus 2% FBS was added onto the cell monolayer, and then the cultures were maintained at 37℃for 5 days. Plaques were visualized by staining

with 1% violet after fixation with 4% formalin.

DNA sequencing and genetic stability analysis of the chimeric viruses

In order to analyze the genome sequence of the chimeric viruses and verify the silent mutation at 473 nt, chimeric viral RNA was extracted and reverse-transcribed to cDNA followed by sequencing. Briefly, total RNA was extracted from the supernatants of infected BHK21 cells using the High Pure Viral RNA Kit (Roche), then reverse transcribed to cDNA using superscript III (invitrogen) and primers 5'-GACTGCTTCCTGTGATTGCA-3' /5'-AGATCCTGTGTTCTTCCTCACCACCAGCTACA-3'. cDNA was amplified with high fidelity enzyme Phusion (NEB) and primers binding to the cDNA. The PCR products were sequenced and analyzed (Invitrogen, Shanghai Branch Company).

Indirect Immunofluorescence Assay (IFA)

To characterize the chimeric JEV/DENV-2 virus, IFA was performed with monoclonal antibodies specific to the DENV-2 E protein (Abcam). The confluent BHK21 cell monolayers in 6-well plates with a 1cm^2 coverslip inside were infected with the chimeric JEV/DENV-2 virus with an M.O.I of 0.05 and propagated for 48 hrs. DENV-2 virus NGC strain and JEV SA14-14-2 were tested as the control viruses. Cells on the coverslips were fixed with 100% methanol, permeabilized with 0.2% Triton X-100, and incubated with primary antibodies (monoclonal antibodies specific for DENV-2 virus E protein, JEV E protein and JEV NS1 protein, respectively, 1:10 dilution) at 37°C for 1 hr. Cells were washed 3 times with phosphate buffered saline (PBS) and then incubated with the secondary antibodies (goat anti-mouse

affinity-purified immunoglobulin G, 1:100 dilution) conjugated with fluorescein isothiocyanate (Abcam) at 37 °C for 30 minutes. The fluorescent signals were visualized using inverted microscope, and images were recorded using a SPOT camera (Olympus).

Viral growth kinetics

Viruses were used to infect C6/36, Vero and primary hamster kindeney (PHK) cells at an M.O.I. of 0.025. Viruses in the supernatants were harvested at an interval of 24 hrs for the C6/36 and Vero cells and at an interval of 12 hrs for the PHK cells. Virus amounts were determined in plaque assays using the BHK21 cells.

Neurovirulence and neuroinvasiveness tests in mice

To assess the neurovirulence of the chimeric virus JEV/DENV-2, groups of 4-week old Kun-Min mice (n=10) were inoculated via the i.c. route with 0.03 ml of ten serial dilutions of the chimeric virus. To determine the neuroinvasiveness, groups of 3-week old Kun-Min mice (n=10) were inoculated via the subcutaneous route with 0.1 ml of ten serial dilutions of the chimeric virus (titre is 5.0 $\log_{10}$PFU). The JE live attenuated vaccine SA14-14-2 was used as the control. Animals were kept for 14 days and the number of deaths was recorded. Moribund animals were euthanized under anesthesia and the $\log_{10}$PFU/LD50 was calculated according to the Reed-Muench method.

Immunogenicity test in mice

Groups of 4-week old Kun-Min mice were immunized via the i.p. route with 0.5 ml of virus (4.3 $\log_{10}$PFU) and boosted with the same amount of virus 2 weeks after the first immunization. Five mice were sacrificed at week 1, 3, 5, 7, 9, 11 after the boost and

sera were collected for subsequent plaque reduction neutralization test (PRNT) to measure the titre of neutralizing antibodies. Briefly, the sera were inactivated at 56℃ for 30 minutes and then diluted at 1:2. The sera were mixed with equal volume DENV-2 virus (NGC strain） of 200 PFU. The serum-virus mixtures were incubated at 37°C for 1 hr before tested in the plaque forming assay. The neutralization antibody titre was determined by the highest dilution that resulted in more than 50% of plaque reduction. The antibody titres from each group were used to calculate the geometric mean titres (GMTs).

Protection against dengue virus and JE virulent virus in mice

To evaluate the protection capacity of the chimeric virus against Dengue virus and JE virulent virus, mice at 4 weeks of age were immunized via the i.p. route with 0.5 ml of the JEV/DENV-2 virus PUO-218 (4.5 log_{10}PFU) and challenged with 0.03 ml of DENV-2 virus NGC strain (4.5 log_{10}PFU) via i.c. route and the JE virulent strain JEV/SA14 (6.6 log_{10}PFU) via the i.p. route 4 weeks later. The JEV/SA14 virus was used as the challenge virus in parallel to detect whether there was any protection against JE virulent virus, because the chimeric virus JEV/DENV-2 was constructed using the JEV SA14-14-2 virus as the backbone and contained all JEV genes except for the prM/E gened from DENV-2.

Statistical analysis

Statistical analysis of mouse mortality rates was carried out using Fisher’s exact test and statistical analysis of the logarithm of 50% PRNT was performed with Analysis of variance (ANOVA). $P<0.05$ was considered statistically significant. All analyses

were performed using SPSS software version 17.0.

Supplementary Figure Legends

Fig. S1 the construction of the recombinant plasmid

Fig. S2 the identification of the recombinant plasmid with restriction enzyme

Lane 1: DNA marker DL15000, lane 2: pACNR-JEV/DENV-2, lane 3: pACNR-JEV/DENV-2 digested with HindⅢ, lane 4: pACNR-JEV/DENV-2 digested with BglⅡ, lane 5: pACNR-JEV/DENV-2 digested with AscⅠ and XhoⅠ, lane 6: pACNR-JEV/DENV-2 digested with BspEⅠ and XhoⅠ, lane 7: DNA marker DL2000.

Supplementary Figure **S1**

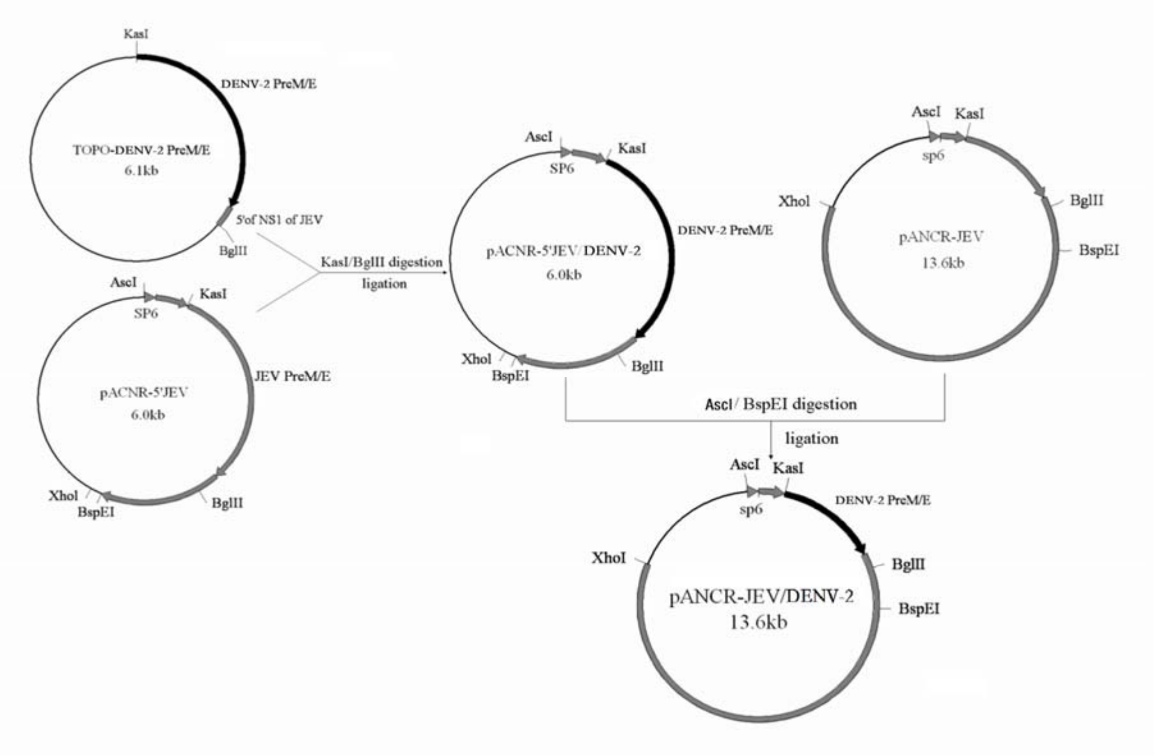

Supplementary Figure **S2**

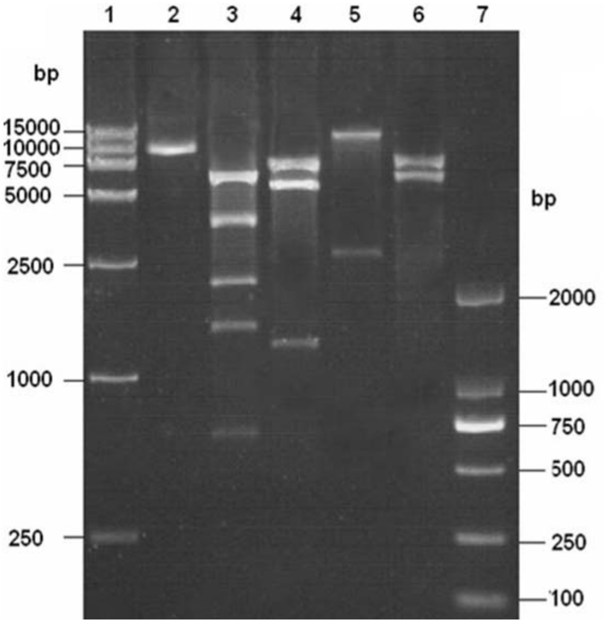

Supplement: Supplementary file 1 — Supplementary material 1 (PDF 127 kb) [file 13238_2016_363_MOESM1_ESM.pdf]
